# High‐Throughput Discovery of Substrate Peptide Sequences for E3 Ubiquitin Ligases Using a cDNA Display Method

**DOI:** 10.1002/cbic.202400617

**Published:** 2024-11-25

**Authors:** Kenwa Tamagawa, Robert E. Campbell, Takuya Terai

**Affiliations:** ^1^ Department of Chemistry Graduate School of Science The University of Tokyo 7-3-1 Hongo Bunkyo-ku, Tokyo 113-0033 Japan

**Keywords:** Degron, Ubiquitylation, cDNA display, E3 ligase, High-throughput screening

## Abstract

Cells utilize ubiquitin as a posttranslational protein modifier to convey various signals such as proteasomal degradation. The dysfunction of ubiquitylation or following proteasomal degradation can give rise to the accumulation and aggregation of improperly ubiquitylated proteins, which is known to be a general causation of many neurodegenerative diseases. Thus, the characterization of substrate peptide sequences of E3 ligases is crucial in biological and pharmaceutical sciences. In this study, we developed a novel high‐throughput screening system for substrate peptide sequences of E3 ligases using a cDNA display method, which enables covalent conjugation between peptide sequences and their corresponding cDNA sequences. First, we focused on the MDM2 E3 ligase and its known peptide substrate as a model to establish the screening method, and confirmed that cDNA display method was compatible with *in vitro* ubiquitylation. Then, we demonstrated identification of MDM2 substrate sequences from random libraries to identify a novel motif (VKFTGGQLA). Bioinformatics analysis of the hit sequences was performed to gain insight about endogenous substrate proteins.

## Graphical abstract

We report a screening system for E3 substrate sequences based on a cDNA display method. A randomized library of potential E3 substrates was displayed in the form of covalent conjugates between the peptide sequences and their corresponding DNA sequences. The displayed library was subjected to *in vitro* ubiquitylation, the ubiquitylated sequences were pulled down by the anti‐ubiquitin antibody, and the corresponding peptide sequences were determined by DNA sequencing.

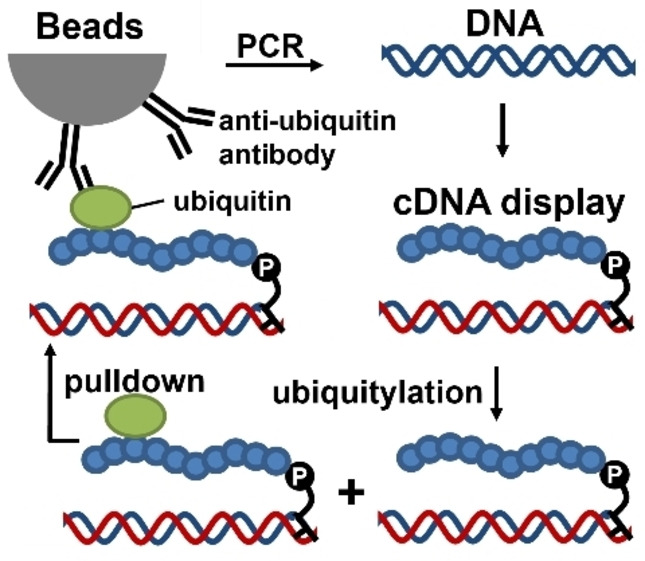

## Introduction

Ubiquitin is a seventy‐six‐amino‐acid small protein and one of the most important posttranslational protein modifiers in eukaryote cellular signaling.[^1^, ^2^, ^3^, ^4^, ^5^, ^6^, ^7^] Ubiquitylation of proteins regulates various biological phenomena such as proteasomal protein degradation,^[3]^ DNA repair,[^8^, ^9^] autophagy^[10]^ and cell cycle progression.^[11]^ Ubiquitylation is accomplished by three enzymatic factors termed E1 ubiquitin activating enzyme, E2 ubiquitin conjugating enzyme and E3 ubiquitin ligase.[^4^, ^6^] In the initial step, E1 activates ubiquitin in an ATP‐dependent manner. The energy originating from the energy‐rich phosphate bond of ATP is preserved in the thioester bond between the C‐terminal carboxyl group of ubiquitin and a thiol group of the internal cysteine residue of E1. Then, the activated ubiquitin is transferred to another cysteine residue of E2 without consuming ATP. Finally, E3 ligases recognize their specific substrates, deliver them to ubiquitin‐conjugating E2, and catalyze the translocation of ubiquitin to the lysine residues of the substrates.

Whereas only two isoforms of E1 and thirty‐eight E2 enzymes were discovered, more than six hundred E3s have been reported in the human genome including some hypothetical proteins. However, it is notoriously difficult identifying E3 ligase substrates because of the high complexity of the E3‐substrate interaction network.^[2]^ Moreover, the physical interactions between E3 ligases and substrates are intrinsically weak and E3‐substrate complexes dissociate rapidly after ubiquitylation in many cases, which makes it hard to co‐precipitate E3‐substrate complexes and identify the interacting substrates. Yet another problem is that the concentration of ubiquitylated substrates is very low in cells due to the obvious reason that they are typically destined to be degraded by the proteasome system.

Several systematic and high‐throughput methods have been developed and utilized to find out E3 ligase substrates for a given E3, or E3 ligases which target a specific substrate in cultured cells.[^2^, ^12^] For instance, global protein stability profiling contributed to the identification of E3 ligase substrates in living mammalian cells.^[13]^ In this technology, substrate candidates of target E3 were fused with a fluorescent protein and the proteasomal degradation of the fused candidates, following ubiquitylation, was detected as a decrease of the fluorescence signal. By analyzing the effect of overexpression or knocking‐down of the target E3, researchers can determine the substrate proteins recognized by the specific E3 ligase. Another example is the ubiquitin ligase trapping method in which the binding affinity between an E3 ligase and its ubiquitylated substrate is increased by fusing a ubiquitin binding domain to the E3.^[14]^ This method enables researchers to overcome the intrinsic low affinity of E3‐substrate conjugates and to pulldown the E3 with its substrates. More recently, E3‐substrate tagging by ubiquitin biotinylation has been developed for sensitive and proximal labeling of substrates.^[15]^ In this method, ubiquitylation is performed using the target E3 fused with a biotin ligase BirA and the ubiquitin possessing a biotin acceptor peptide in order to obtain substrate proteins that are both biotinylated and ubiquitylated, which are then subjected to a pulldown procedure.

*In vitro* ubiquitylation assays using protein microarrays can provide a simpler way of defining E3 substrates.^[16]^ This method uses a recombinant protein library immobilized on a carrier surface, such as microscope glass slide, and subjects them to the reconstituted ubiquitylation system. In order to detect the ubiquitylated substrates, a fluorescent label is attached to ubiquitin beforehand or labeled ubiquitin probes are used such as anti‐ubiquitin antibody and the tandem ubiquitin‐binding entity.^[17]^

Although there have been many efforts to develop high‐throughput methods to identify E3 substrates as described above, these methods often suffer from laborious library preparation or limited library size. For example, the maximum library size available on a protein microarray is around 10,000 proteins because each single protein must be expressed and purified independently. The global protein stability profiling also requires time‐consuming cloning of each protein's gene into the reporter gene. Furthermore, the existing methods are whole protein‐based and may require additional experiments to identify the recognition peptide sequences of E3, which are called degrons. Moreover, they only screen endogenous proteins and miss the huge sequence space of unexplored degrons that may be more efficient or specific compared with natural ones. Obtaining such information should be potentially useful to develop new therapeutic technologies and tools for biological studies.[^18^, ^19^]

**Figure 1:**
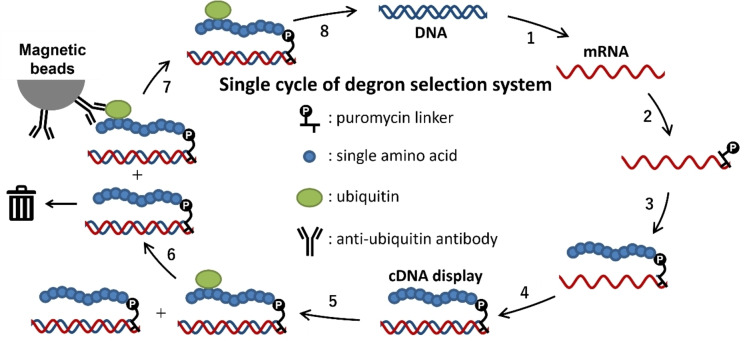
Scheme of cDNA display‐based selection of degron sequences. All steps of the degron selection are performed *in vitro* manner. The selection cycle starts from transcription of DNA library to obtain mRNA library (step 1). Next, a puromycin liker (Figure S1) is attached to the 3’ terminus of the mRNA by photo‐crosslinking reaction (step 2; see Figure S2 and S3) which achieves covalent conjugation of a peptide and the corresponding mRNA in the subsequent translation (step 3). Then, reverse transcription is performed to copy the genetic information of mRNA to cDNA (step 4). The obtained cDNA display sample is subjected to ubiquitylation reaction (step 5), and only the ubiquitylated display molecules are pulled down by the anti‐ubiquitin antibodies and then eluted (step 6, 7). Finally, the eluent from the pulldown is amplified by PCR to get the DNA sample for the next round (step 8).

Herein, in order to develop a novel screening method to seek out the degron sequences from a large *in vitro* designed peptide library, we aimed to exploit the advantages of the cDNA display method.[^20^, ^21^, ^22^, ^23^] This is one of the display technologies that involve the formation of covalent conjugates between peptides and their corresponding nucleic acid sequences (Figure 1). In the first part of the selection, a randomized DNA library is converted to the cDNA display library using a synthetic DNA linker containing puromycin by an *in vitro* transcription and translation system. The obtained cDNA display library is then subjected to E3‐catalyzed ubiquitylation, and the ubiquitylated display molecules are pulled down with the magnetic beads conjugated with anti‐ubiquitin antibodies. The cDNA display construction and screening are repeated for several rounds in order to concentrate more preferred substrate sequences from the library. After several rounds of selection, the selected sequences are read out and analyzed by next‐generation sequencing.

## Results and Discussion

### Ubiquitylation of cDNA Displayed p53 Degron

**Figure 2:**
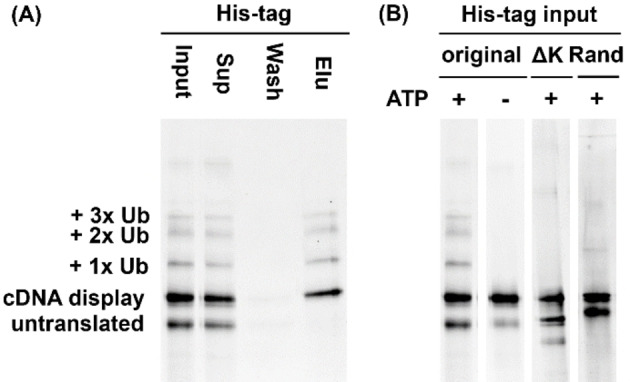
*In vitro* ubiquitylation of cDNA display molecules. (A) SDS‐PAGE of the p53deg display samples collected during the His‐tag purification. The bands were visualized by FITC chemically attached to the puromycin linker (Figure S1). (B) His‐tag input samples of the control experiments. The ATP+ lane of the original p53deg (1^st^ lane) is the same with the input lane of (A).

As the initial step of the study, we examined if cDNA display molecules incorporating a known degron was ubiquitylated *in vitro* as expected. We used MDM2, one of the RING‐typed E3 ligases, and p53 substrate pair as a model system. The twenty‐one‐amino‐acid‐length degron previously found from p53 (PLSSSVPSQKTYQGSYGFRLG)[^24^, ^25^] was expressed as cDNA display molecule with addition of several lysine residues for enhancement of ubiquitylation and the histidine tag at the C‐terminus (named p53deg; Table 1). The cDNA display sample was prepared according to the standard protocol^[21]^ and subjected to a reconstituted ubiquitylation system which consisted of UBA1 (E1), UbcH5c (E2), and MDM2 (E3). Then, the ubiquitylated product (and non‐ubiquitylated cDNA display) was purified by Ni^2+^ supporting beads since the direct pulldown from the ubiquitylation reaction mixture can be hampered by the excess amount of free ubiquitin in the actual selection. The samples collected during the histidine‐tag purification were analyzed by SDS‐PAGE. Figure 2A shows that bands attributable to mono‐, di‐ and tri‐ubiquitylated display molecules appeared and were purified together with non‐ubiquitylated displays (=remaining substrate). On the other hand, the untranslated molecules, which are cDNA without any peptide, were removed as they did not have the histidine tag.

**Table 1:** The sequences of the displayed peptides. The residue X represents the random residue translated from the NNK codon. The difference from p53deg and randomized part are highlighted in bold underline. “His‐tag” in the sequences represents GGGSHHHHHHGGS sequence.

Name	Amino acid sequence
p53deg	KPLSSSVPSQKTYQGSYGFRLGKKKK+His‐tag
p53degΔK	**A** PLSSSVPSQ **A** TYQGSYGFRLG **AGGS** +His‐tag
p53degRand	K **XXXXXXXXXXXXXXXXXXXXX** KKKK+His‐tag
p53deg library	KPLSSSVPSQ **XXXXXXXX** FRLGKKKK+His‐tag
LX9 library	AGGS **XXXXXXXXX** GGS+His‐tag

Two variants of p53deg, named p53degΔK and p53degRand (Table 1), were also displayed and ubiquitylated in the same manner. p53degΔK was derived from the original p53deg replacing all lysine residues with other residues. As for p53degRand, the whole degron part was replaced by consecutive NNK codons while keeping its original length. These variants showed only two major bands which corresponded to the untranslated linker conjugate and un‐ubiquitylated cDNA display (Figure 2B, 3^rd^ and 4^th^ lane). As another negative control experiment, ubiquitylation of the original p53deg was carried out in the absence of ATP to test the ATP dependency of ubiquitylation of the display molecules (Figure 2B, 2^nd^ lane). These results indicate that when the degron sequence is appropriately displayed on its cDNA, it is recognized as a substrate of E3 and ubiquitylated.

### Model Selection Using p53 Degron

**Figure 3:**
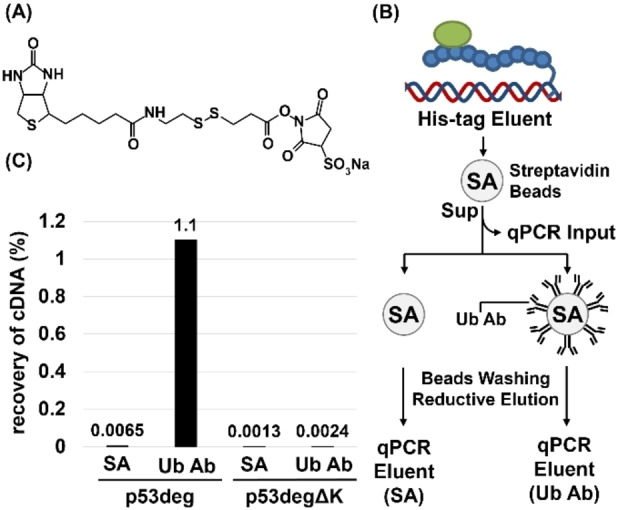
Model selection of p53 degron using antibody pulldown. (A) The chemical structure of the biotinylating reagent used for the antibody biotinylation. (B) The scheme of the model selection of the ubiquitylated display molecules. (Sup: supernatant, SA: streptavidin, Ub Ab: anti‐ubiquitin antibody) (C) The recovery of cDNA during the pulldown.

Next, we worked on purification of the successfully ubiquitylated cDNA display molecules from the sample. Here, cDNA display of p53deg was subjected to pulldown by the magnetic beads conjugated with the anti‐ubiquitin antibody. The antibody was biotinylated using an NHS ester‐type biotinylating reagent (Figure 3A) and immobilized on streptavidin beads beforehand. We used a reagent with a disulfide bond in the linker region that allowed for reductive elution of the antibody from streptavidin beads later.

After *in vitro* ubiquitylation of non‐purified cDNA display (the mixture of correctly formed cDNA display and the untranslated cDNA without any peptide) and the subsequent His‐tag purification to remove free ubiquitin and enzymes, the eluent from the Ni^2+^ supporting beads was subjected to negative selection using unprocessed streptavidin beads (Figure 3B). The supernatant was collected and a small portion was kept as a standard for qPCR analysis (=“input”). The remaining sample was used for the next positive selection. Specifically, half of the sample was incubated with antibody conjugated beads, and after several washes, the ubiquitylated products were eluted from the beads using tris(2‐carboxyethyl)phosphine (TCEP) as a reductant to cleave disulfide bonds of the antibody as well as that in the linker (=“Ub Ab” elute). As a comparison, the other half of the sample was incubated with intact streptavidin beads again and subjected to the same procedure (=“SA” elute). The DNA contained in the two eluents were then quantified by qPCR, and the recovery value was calculated relative to the input.

For p53deg, the recovery for the control streptavidin beads was 6.5×10^‐3^ %, while that for the antibody beads was 1.1 %, which suggests that the ubiquitin attached on the displayed peptide is properly recognized by the antibody (Figure 3C). As another negative control, the recovery of p53degΔK was measured in the same way with the original degron. The value was 1.3×10^‐3^ % for the control beads and 2.4×10^‐3^ % for the antibody conjugating beads, which did not show significant difference. Together with Figure 2(B), the result suggests that the substantially increased recovery of the Ub Ab elute of p53deg does not come from the nonspecific binding between the cDNA display and the anti‐ubiquitin antibody but from the interaction of ubiquitylated cDNA display molecules and the antibody.

Overall, these experiments confirmed that the cDNA display method was compatible with *in vitro* ubiquitylation and the ubiquitylated cDNA displayed degron could be selectively pulled down by the anti‐ubiquitin antibody beads as illustrated in Figure 1.

### Actual Screening of MDM2 Degron Sequences

Having successfully performed the model selection using p53 degron, we moved on to the screening of degron sequences for MDM2 from a randomized library. First, we prepared a partially randomized p53 degron sequence, named p53deg library, where eight consecutive residues were randomized from the original p53deg (Table 1).

The library underwent five successive rounds of selection. In the initial round, 20 pmol of mRNA, which is theoretically sufficient to include all of the possible sequence patterns of eight NNK codons, were converted to cDNA display, incubated in ubiquitylation reaction mixture, His‐tag purified, and subjected to the positive selection and negative selection. The eluent from the beads was PCR amplified and the obtained DNA was used as the template for the next round. From the 2^nd^ to the 5^th^ rounds, the selection was performed by applying 10 pmol of mRNA. To evaluate the enrichment, the initial library and the selected library (=the eluent of the 5^th^ round) were separately displayed, ubiquitylated and the cDNA recovery was measured by the pulldown assay as described above. Simultaneously, another cDNA display samples from the two libraries were prepared and subjected to ubiquitylation without ATP. These samples underwent the pulldown assay in the same manner. Based on the direct recoveries for ATP‐positive and negative conditions, the recovery ratio was calculated by dividing the recovery in ATP‐positive condition by that in ATP‐negative condition (see Supporting Information for details).

**Figure 4:**
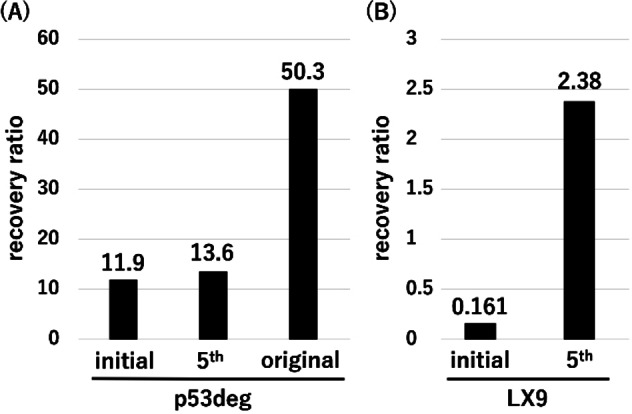
Recovery ratio of the initial and the 5^th^ round samples from selection against the p53deg library (A) and the LX9 library (B). The “original” lane shows the recovery ratio of the original p53deg sequence.

Contrary to our expectations, there was no substantial difference observed in the recovery ratio between the initial library and the library after the 5^th^ round of selection when we started from the p53deg library (Figure 4, left). It was also confirmed that the recovery ratio of the 5^th^ round sample was far less than that of the original p53deg sequence. This result suggests that the remaining degron sequence and the additional lysine residues likely facilitated ubiquitylation to an extent that the recovery ratio of the initial library was already high for a starting point. Overall, the selection using p53deg library resulted in insufficient amplification of functional sequences probably due to the excessive background ubiquitylation resulting from the inappropriate library design.

Making use of the identified issues in library design during the selection using the p53deg library, a new DNA library was created, consisting of nine consecutive NNK codons without additional lysine residues (referred to as LX9 library; Table 1). In the initial round, 60 pmol of mRNA were applied for the selection. This new library underwent five rounds of selection in total following a similar protocol as the one applied to the p53deg library. Then, the recovery ratio of the initial library and the library from the 5^th^ round was measured using the same protocol.

The results revealed a notably higher recovery ratio for the 5th round's library compared to the initial library (Figure 4, right). Ideally, the recovery ratio for the initial round should be around one due to the presence of a slight amount of the substrate sequence in the initial library. However, experimental data indicated a significantly lower recovery ratio for the initial library than expected. As the recovery ratio is defined as the recovery in ATP‐positive conditions divided by that in ATP‐negative conditions, the result suggests that either the presence of ATP reduces recovery in this experimental design or the absence of ATP enhances recovery, but the exact mechanism remains unclear. Despite this uncertainty, it is evident that the 5^th^ round's sample exhibited a much higher recovery ratio than the initial library. Consequently, the sequence composition of the sample was analyzed using next‐generation sequencing.

### Next‐Generation Sequencing

The sequence composition of the 5^th^ round's sample of the selected LX9 library underwent analysis using the MiSeq (Illumina) next‐generation sequencing system. This analysis revealed 8,548 unique sequences among a total read count of 136,664. Unexpectedly, it was revealed that the majority of the top sequences lacked lysine residues, with the first lysine‐containing sequence appearing at the 8^th^ ranked position (in order of abundance, see Table S1). This suggests that another selection pressure rather than the expected ubiquitylation was present in the selection. We also sequenced the 5^th^ round's sample of p53deg library, but again, most of the top sequences did not have any lysine residues (Table S2 and supplementary text).

One possible explanation for this result is that MDM2 binding peptides were amplified during the selection process. Given MDM2′s intrinsic self‐ubiquitylation ability, which is known to enhance the ligase activity by facilitating more efficient recruitment of E2 through the non‐covalent interaction between ubiquitin on MDM2 and the ubiquitin binding domain of E2,^[26]^ MDM2 binding peptides may have been pulled down by anti‐ubiquitin antibody as a complex with ubiquitylated MDM2. Another possible explanation is that peptides that bind to the anti‐ubiquitin antibody were selected, because the negative selection was performed using non‐conjugated SA beads.

To focus on only those peptides that are potential substrates for ubiquitylation, a table of sequences containing one or more lysine residues was established and the top four highest‐ranking lysine‐containing sequences were cloned for further characterization (Table 2).

**Table 2:** Top 4 lysine containing sequences discovered by the selection using LX9 library.

Rank	Name	Amino acid sequence (randomized region)	Recovery ratio	BLAST entries
8	LX9K1	**K** HVCVGWLV	3.03	DNAJB6 (*Harpia harpyja*)
16	LX9K2	GR **K** SRHWSD	0.415	2 unnamed proteins
21	LX9K3	V **K** FTGGQLA	18.4	2 unnamed proteins
27	LX9K4	PCARS **K** HWS	4.08	Proteasome α1 subunit (*Symbiodinium microadriaticum*), 1 unnamed protein

### Characterization of the Discovered Sequences

The full‐length DNA of the top four lysine‐containing sequences (LX9K1‐LX9K4, Table 2), encompassing the necessary DNA motifs for cDNA display construction, were prepared using the same methodology as employed for the library. Subsequently, these samples underwent cDNA display construction, ubiquitylation, and pulldown assays following identical procedures to those of the selection process. The recovery ratio for each variant is provided in Table 2. Among the four tested sequences, three exhibited higher recovery ratios than that of the 5^th^ round's library (2.38, as shown in Figure 4), with LX9K3 showing a significantly elevated value of 18.4 (Table 2). It is important to note that the 5^th^ round library includes several non‐functional sequences, and so the averaged recovery ratio of the library tends to be smaller than that of a single clone.

**Figure 5:**
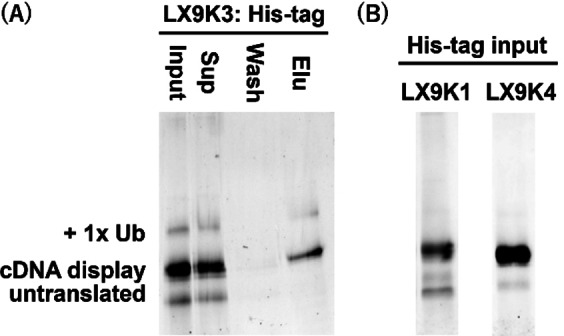
The results of *in vitro* ubiquitylation of the discovered sequences. (A) SDS‐PAGE of the displayed LX9K3 samples collected during the His‐tag purification. The bands were visualized by FITC attached to the puromycin linker. (B) His‐tag input samples of LX9K1 and LX9K4 sequences.

In order to directly confirm whether the discovered sequences are ubiquitylated by MDM2, we constructed the cDNA displays of LX9K1, LX9K3, and LX9K4 sequences and performed *in vitro* ubiquitylation. We then analyzed the results using SDS‐PAGE, following the same protocol previously applied to p53deg (see Figure 2). LX9K3, which had the highest recovery ratio among the three peptides, was validated as an MDM2 substrate, as indicated by the appearance of an upper band above the band of cDNA display molecules (Figure 5A). In contrast, the other two sequences did not show any bands for ubiquitylation (Figure 5B). Since the upper band in the input lane of Figure 5A also appeared in the elution lane, we concluded that this band corresponds to mono‐ubiquitylated cDNA display molecules.

Considering that LX9K1 and LX9K4 exhibited more than a 10‐fold higher recovery ratio than the initial LX9 library, these sequences may be binders to MDM2 or the anti‐ubiquitin antibody, as described in the previous section. Another possible explanation is that these sequences are weak MDM2 substrates, so the ubiquitylated display molecules could not be visualized by fluorescent detection of the linker due to very small amounts of the products.

### BLAST Analysis

The identified sequences from the selection were analyzed using the BLAST online interface to identify naturally occurring proteins (if any) with similar sequences. The top four sequences containing lysine residue were first scrutinized. Most of the identified proteins were hypothetical proteins present in various bacterial or fungal genomes. However, these are unlikely to serve as endogenous substrates (or binders) for MDM2, considering that the oldest MDM2 originated from multicellular eukaryotic organisms and proliferated in animals.[^27^, ^28^] Since we applied human MDM2 in the selection, we first limited the analysis to human proteins but no hits were found. Thus, we expanded the analysis to all eukaryotes, although it is not necessarily true that their MDM2 have the same specificity as the human MDM2. Please also note that we do not claim that the identified entries should actually interact with human MDM2 but rather highlight the potential bioinformatic application of randomized library‐based degron screening.

Interestingly, two isoforms of DnaJB6 (DnaJ homolog subfamily B member 6; NCBI Reference Sequence: XP 052662674.1 and XP 052662682.1) from *Harpia harpyja* (eagles) were found to harbor the HVCVGWL motif, akin to LX9K1 (Table 2). The DnaJ family, also known as the heat shock protein family (Hsp40), is associated with protein folding and the assembly of oligomeric protein complexes. It is noteworthy that another member of the DnaJ family, DnaJB1, was previously reported to interact with MDM2, preventing MDM2‐catalyzed ubiquitylation and subsequent proteasomal degradation of p53.^[29]^ This suggests a potential interaction between DnaJB6 and MDM2.

Another noteworthy finding was a sequence in the α1 subunit of the proteasome (GenBank: OLQ03828.1) from *Symbiodinium microadriaticum* (symbiotic algae in coral) (Table 2). We also confirmed that this organism has a protein homologous to human MDM2 (GenBank: CAE7256442.1), identified with an E‐value of 1×10^−9^ through BLAST search against human MDM2. It was previously reported that the direct interaction between the 19S proteasome regulatory particle and MDM2 itself resulted in ubiquitin‐independent p53 degradation.^[30]^ Moreover, interaction between MDM2 and the α7 subunit (also known as C8) of 20S core particle was reported to facilitate ubiquitin‐independent degradation of p21, another factor of cell cycle regulation, apoptosis and gene transcription after DNA damage.[^31^, ^32^] Considering the previously revealed direct interactions between MDM2 and proteasome components, the discovered LX9K4‐like sequence may imply a potential interaction between MDM2 and α1 subunit of the proteasome.

## Conclusions

A novel E3 ligase substrate screening system using the cDNA display method was developed in this study. In the initial part of the study, each step of the screening system was examined using the MDM2 E3 ligase and p53 substrate pair. It was confirmed that the degron of p53 was compatible with the cDNA display method and that the displayed peptide was recognized by MDM2 and ubiquitylated as expected. The ubiquitylated cDNA displayed degron was selectively pulled down by the anti‐ubiquitin antibody beads, and much higher cDNA recovery was observed compared with the control degron without lysine residues.

In the next step, screening of MDM2 degron from a randomized library was performed on the established system as a proof of principle. The selection using a library composed of nine random residues and the following *in vitro* ubiquitylation assay resulted in the discovery of a novel substrate motif of MDM2, VKFTGGQLA. Two other potential substrate motifs or binders were selected, too, which showed higher recovery ratio than the selected library. BLAST analysis revealed that a member of heat shock protein family and a subunit of proteasome, which are likely to be related to ubiquitylation pathway, possess similar motifs.

Although *in vitro* display technologies including cDNA display have been extensively applied to the selection of peptide binders against target proteins,[^20^, ^21^, ^33^, ^34^] the scope of these technologies is not limited to binder selection. For example, Tsuboyama and coworkers recently reported a cDNA display‐based method for analyzing thermostability of proteins.^[35]^ Also, Chang *et. al* reported the profiling of Laz enzymes’ substrates using mRNA display,^[36]^ and Nakano and others reported substrate profiling of transglutaminase 1 using cDNA display.^[37]^ To the best of our knowledge, this study is the first report of cDNA display for screening of substrates of E3 enzymes. Although our screening platform does not distinguish true substrates from some kinds of false positives such as ubiquitin binders, we can perform a secondary screening to identify novel substrates as shown in Figure 5.

As a future direction, the efficacy of the discovered peptide as a degron when fused to a protein of interest, will need to be investigated. In order to effectively target the protein for degradation, many conditions such as the length and composition of the linker connecting the degron and the protein, would need to be optimized. In principle, this selection method could be extended for other classes of E3 ubiquitin ligases such as multi‐subunit RING‐typed,^[38]^ HECT‐typed^[39]^ or RBR‐typed^[40]^ E3 in order to demonstrate general applicability of the system. One possible further application is degron screening for E3 ligases that are overexpressed in a tissue‐ or disease‐specific manner. In such cases, the identified degron fused to a protein that binds specifically to the target protein (e. g., a nanobody^[41]^), could be used as a genetically encoded degradation tag of the target proteins in the specific tissues or disease conditions.

## Supporting Information Summary

Detailed information about the puromycin linker, photo‐crosslinking reaction, sequencing results, additional analysis on discovered sequences and experimental procedures are available in the Supporting Information. The authors have cited additional references within the Supporting Information.[^42^, ^43^, ^44^, ^45^]

## Conflict of Interests

The authors declare no conflict of interest.

1

## Supporting information

As a service to our authors and readers, this journal provides supporting information supplied by the authors. Such materials are peer reviewed and may be re‐organized for online delivery, but are not copy‐edited or typeset. Technical support issues arising from supporting information (other than missing files) should be addressed to the authors.

Supporting Information

## Data Availability

The ensembled NGS data for the 5^th^ round's samples of both p53deg and LX9 libraries are available in external repository (https://zenodo.org/records/13957071).^[46]^ The top 50 sequences of each selection are also summarized in Table S3 and S4. Other data that support the findings of this study are available from the corresponding author upon reasonable request.
