# Supplementary material for: High‐Throughput Discovery of Substrate Peptide Sequences for E3 Ubiquitin Ligases Using a cDNA Display Method
Source: Chembiochem. 2024 Nov 25;25(24):e202400617. doi: 10.1002/cbic.202400617 (PMC11664913; doi:10.1002/cbic.202400617)
Supplement: Supplementary file 1 — Supporting Information [file CBIC-25-e202400617-s001.pdf]

ChemBioChem

Supporting Information

High-Throughput Discovery of Substrate Peptide Sequences for E3 Ubiquitin Ligases Using a cDNA Display Method

Kenwa Tamagawa, Robert E. Campbell, and Takuya Terai*

High-Throughput Discovery of Substrate Peptide Sequences for E3 Ubiquitin Ligases Using a cDNA Display Method

Kenwa Tamagawa,[a] Robert E. Campbell[a], and Takuya Terai*[a]

[a] K. Tamagawa, Prof. R. E. Campbell, Dr. T. Terai
Department of Chemistry, Graduate School of Science
The University of Tokyo
7-3-1 Hongo, Bunkyo-ku, 113-0033 Tokyo, Japan
E-mail: terai@chem.s.u-tokyo.ac.jp (T.T.)

Supporting Information

Table of contents

Figure S1. Structure of the puromycin linker	p. 2
Figure S2. Structure of ^{cnv}K base and the mechanism of photo-crosslinking reaction	p. 3
Figure S3 A representative result of photo-crosslinking reaction	p. 4
Table S1. Top 10 sequences discovered by the selection using LX9 library	p. 5
Table S2. Top 10 sequences discovered by the selection using p53deg library	p. 6
Table S3. Top 50 amino acid sequences with their read count from LX9 library.	p. 7
Table S4. Top 50 amino acid sequences with their read count from p53deg library.	p. 8
Supplementary text	p. 9
Experimental section	p. 11
List of primer sequences	p. 18

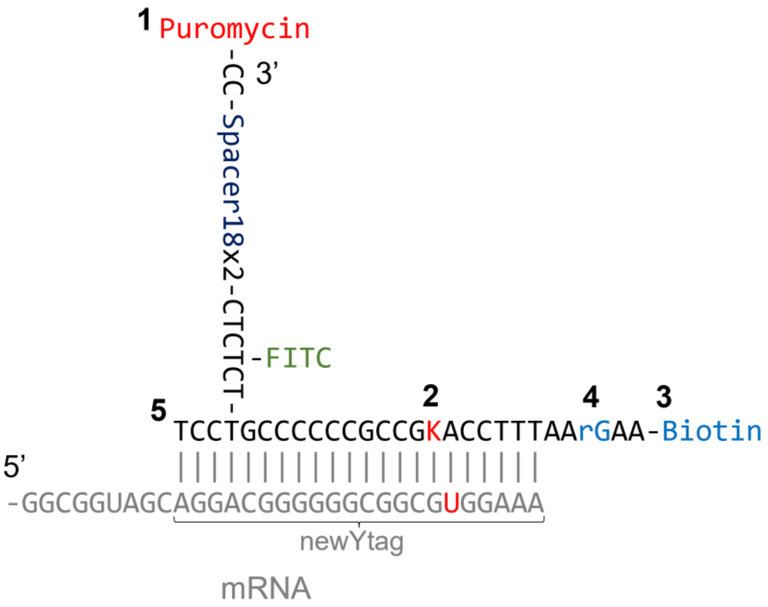

Figure S1. Structure of the puromycin linker.[42] The linker contains several important groups for further processing. Puromycin on the top of the linker (**1**) is used for conjugation with the peptide. ^{cnv}K (3-cyanovinylcarbazole nucleoside)[43] in the DNA scaffold (**2**) is an unnatural base developed for ultrafast reversible interstrand photo-crosslinking reactions. This base can be incorporated in DNA sequences as a nucleotide and forms four-membered ring with pyrimidine bases such as C, T or U in the counter strand when it is irradiated with 365nm UV light. Biotin (**3**) is attached for the purification after in vitro translation using streptavidin-coated beads, and guanine ribonucleotide (**4**) is for elution from the streptavidin beads using RNaseT1. The primer region for reverse transcription is provided by linker (**5**).

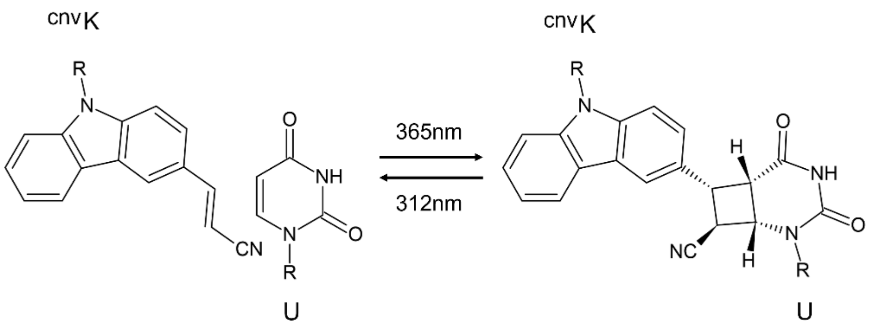

Figure S2. Structure of ^{cnv}K base and the mechanism of photo-crosslinking reaction with an uracil in the complementary strand.

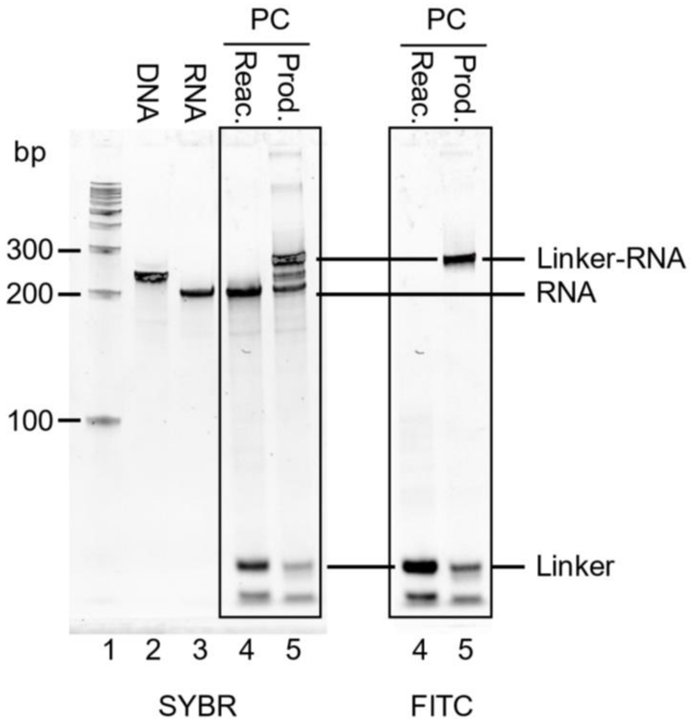

Figure S3. A representative result of transcription and photo-crosslinking reaction of p53deg sequence. The picture in the right-hand side was taken with FITC attached to the linker. The picture in the left-hand side was taken after staining with SYBR Gold so that nucleotide which did not have FITC were visualized. The boxed areas correspond to the same region on the gel. Loaded samples: lane 1: DNA ladder (100 bp to 1000 bp with 100 bp step and 1500 bp), lane 2: DNA of p53deg (235 bp), lane 3: RNA of p53deg (205 mer), lane 4: The reaction mixture of photo-crosslinking reaction before UV irradiation, lane 5: The photo-crosslinking reaction product.

Table S1. Top 10 sequences discovered by the selection using LX9 library.

Rank	Name	Amino acid sequence	Proportion [%]
1	LX9N1	GQQALQADA	0.88
2	LX9N2	GRSMRYVRD	0.68
3	LX9N3	SWIVAGVSG	0.19
4	LX9N4	RWRCLDRQA	0.18
5	LX9N5	VQGWCSHAR	0.14
6	LX9N6	VLVNDLCVF	0.13
7	LX9N7	AMAVLCALT	0.13
8	LX9N8 (= LX9K1)	**K**HVCVGWLV	0.13
9	LX9N9	VRWGCMCVA	0.11
10	LX9N10	QNRASIGCS	0.10

Table S2. Top 10 sequences discovered by the selection using p53deg library.

Rank	Name	Amino acid sequence	Proportion [%]	Recovery ratio
1	p53lib-1	TAGGLNAQ	0.15	8.93
2	p53lib-2	DVEYDPSD	0.14	4.54
3	p53lib-3	GVSLTAGG	0.11	N/A
4*	p53lib-4 (= p53deg)	**K**TYQGSYG	<0.1	50.1
5	p53lib-5	IGENEFYG	<0.1	-
6	p53lib-6	FNGALL**K**V	<0.1	-
7	p53lib-7	STGENTGV	<0.1	-
8	p53lib-8	IYAESQGA	<0.1	-
9	p53lib-9	NHPGSTAI	<0.1	-
10	p53lib-10	LTYSSEEL	<0.1	-

*Maybe a contamination from the positive control. See supplementary text.

Table S3. Top 50 amino acid sequences with their read count from LX9 library. The total read count was 136,664. The asterisk marks in the sequences represent the top codons.

Rank	Sequence	Count	Rank	Sequence	Count
1	GQQALQADA	1200	26	MSRVRLPPM	105
2	GRSMRYVRD	934	27	PCARSKHWS	104
3	SWIVAGVSG	253	28	VCRAEYAPR	104
4	RWRCLDRQA	248	29	LPVGQQGIP	103
5	VQGWCSHAR	192	30	GVTECRGHD	102
6	VLVNDLCVF	183	31	AYSAMDYRL	101
7	AMAVLCALT	180	32	TGKVCCGTE	101
8	KHVCVGWLV	179	33	GRWHAYVSV	100
9	VRWGCMCVA	148	34	VQLRWRRSG	97
10	QNRASIGCS	138	35	ARRVLVMYT	96
11	MDGWLGSCA	137	36	TKSAICVRI	96
12	ASRRDRSAV	136	37	GGPVHSCGS	95
13	*ARPYQCCS	126	38	HRVNHRLRP	94
14	AAIRRAYNL	121	39	CASIPCNFA	91
15	VRRDRPPAD	119	40	ESDGGITGQ	90
16	GRKSRHWSD	118	41	RHGHGGLCG	89
17	GACDVHRDI	117	42	AGWSERSRV	89
18	VPWRLPYVS	115	43	CAGRTFASV	88
19	CPVPHRTVL	114	44	AVCTVLREG	88
20	LQCMHGDGP	112	45	VLFWTVASM	88
21	VKFTGGQLA	111	46	GVR*TGETG	86
22	IVRIRRACG	110	47	SRLNSARVH	86
23	GAVVSRMLG	110	48	RPTYGATAG	86
24	RLVGWFMWR	109	49	RRACKNPLV	85
25	GVGWW*SQP	106	50	TQASAYRWC	84

Table S4. Top 50 amino acid sequences with their read count from p53deg library. The total read count was 118,484. The asterisk marks in the sequences represent the top codons.

Rank	Sequence	Count	Rank	Sequence	Count
1	TAGGLNAQ	177	26	DVGGVGMM	48
2	DVEYDPSD	163	27	LAAKELGV	45
3	GVSLTAGG	130	28	PVRGRIRA	43
4	KTYQGSYG	111	29	HVIVAMEI	43
5	IGENEFYG	83	30	GDEMNVDI	43
6	FNGALLKV	75	31	YDEDAWVG	43
7	IYAESQGA	74	32	VTPGMISW	42
8	STGENTGV	73	33	VH*VSTAV	42
9	NHPGSTAI	72	34	SVMMLMTM	39
10	LTYSSEEL	71	35	HQLVYSAC	39
11	RFYVPAED	71	36	G*RQMMRG	39
12	HEGVNDQD	67	37	SMNGTDAY	38
13	DLNAAQLS	64	38	ADVLAVAL	38
14	DAFLHTQF	63	39	RTGVVVGI	38
15	DVLEQCQV	63	40	TVTQPLIQ	38
16	LDLVDSMA	63	41	SGQEAVTP	37
17	SASTCSTN	60	42	FPRLMTLT	37
18	TLLWEYEV	59	43	SVRQHCGG	37
19	YDDYAGEV	57	44	AGRIRSA*	37
20	LEYVLDMP	55	45	IDMDDTTI	37
21	TRYIGMAA	55	46	LAAPSVRL	36
22	AAMSVPLL	55	47	T*GLLRKR	36
23	QWLSGSAW	53	48	VG*WRGLG	35
24	VILPEEQW	50	49	SQCVCYVN	35
25	CHLHAPGA	49	50	YEMVSELH	35

Supplementary text

Analysis for the selection using p53deg library

In a parallel effort with the analysis of the selected LX9 library, the sequence composition for the selection using the p53deg library was also analysed by the next generation sequencing although this library did not show substantial increase in recovery ratio. As a result, 52,804 unique sequences were discovered in a total read count of 118,484. The larger number of the unique sequences, compared to the case of the LX9 library (8,548), corresponds with the insufficient amplification of functional sequences during the selection. Surprisingly, the KTYQGSYG motif (Table S2), which perfectly matches the original p53 degron, appeared in the 4th rank. However, the DNA sequence of this amplicon was exactly the same with a sample that was used in other control experiment and there were no synonymous codons in the re-discovered p53 degron sequence. Therefore, we concluded that this sequence was probably contaminated during the selection and amplified to some extent until the final round.

The top four sequences out from the p53deg library, including the original p53 degron, were cloned, displayed and analyzed by the pulldown assay (Table S2). It was, however, revealed that none of the tested sequences, except for the original degron, exhibited higher recovery ratio than the original p53deg 5th round library. It can be said that the sequences appeared in the top population are non-functional and amplified in a stochastic manner.

BLAST analysis for the discovered sequences other than LX9K1 and LX9K4

The analysis revealed that a hypothetical protein (GenBank: KAJ1096018.1) from *Megalop atlanticus* (bony fishes) and another hypothetical protein (GenBank: KAJ1096018.1) from *Pleurodeles waltl* (salamanders) contain motifs similar to LX9K2 (RKSRHWAD and RKSRHWSE, respectively).

For LX9K3 sequence, which showed the highest recovery ratio, a hypothetical protein (GenBank: RLW03009.1) from *Chloebia gouldiae* (gouldian finch) possesses the VKFTGGELA motif in its N-terminus, while an uncharacterized protein (NCBI Reference Sequence: XP_053677197.1) from *Anopheles nili* (mosquito) carries the VKFTEGQLA motif in its near N-terminus. Although there are no reported functionalities for these two proteins, they have the potential to be ubiquitylated by MDM2 or interact with MDM2.

Similarly, the top four sequences lacking lysine residues were examined with the hope of gaining insights into these sequences. These sequences were designated as LX9N1, LX9N2, LX9N3, and LX9N4, corresponding to their respective rankings. While there were no registered eukaryotic proteins containing LX9N1-like motifs, a hypothetical protein (GenBank: KAH0821660.1) from *Tenebrio molitor* (yellow mealworms) and a phenylalanine 2-monooxygenase precursor-like protein (NCBI Reference Sequence: XP_037050894.1) from *Bradysia coprophila* (flies) were discovered to have a

motif similar to LX9N2. UPF0462 protein C4orf33 homolog isoform X1 (NCBI Reference Sequence: XP_005104524.1) from *Aplysia californica* (sea hares) has the SWIVSGVSG motif, which is similar to LX9N3 in its near C-terminus. Finally, the examination of the LX9N4 motif resulted in the discovery of more than one hundred entries of putative nuclease HARBI1 from various kinds of bony fishes. HARBI1 is a transposon-derived protein conserved in various bony fishes to humans.[44,45] The nuclease activity of HARBI1 is expected to be important; however, its exact role is still uncharacterized.

Experimental section

Primer overlap extension reaction

Three DNA oligonucleotides purchased from Invitrogen™, named T7PURE, p53_deg, and KHis_cnvK were connected into a 235 bp length DNA named p53deg by two runs of primer overlap extension using PrimeSTAR HS DNA Polymerase system (TOYOBO, product number: R101A). 50 μL of reaction mixture for the first extension contained 10 μL of 5×PrimeSTAR buffer, 4 μL of 2.5 mM each dNTP mix solution, 5 μL of 10 μM p53_deg solution, 5 μL of 10 μM K4_His_cnvK solution, 0.5 μL of PrimeSTAR polymerase and 25.5 μL UPDW (UltraPure™ DNase/RNase-Free Distilled Water, Invitrogen™, product number: 10977015). The mixture was heated by thermal cycler (Biometra TRIO 48, analytik jena, product number: 846-5-070-723) as following the recommended thermal cycler's program described by the manufacturer of the polymerase. The product DNA was purified by FavorPrep™ GEL/PCR Purification Kit (FavoGene Biotech Corp., product number: FAGCK 001) following the instruction of the kit. Reaction mixture for the second extension contained 10 μL of 5× PrimeSTAR buffer, 4 μL of 2.5mM each dNTP mix solution, 3.75 μL of 10 μM T7_PURE solution, 30 μL of first extension product solution, 0.5 μL of PrimeSTAR polymerase and 1.75 μL UPDW. The mixture was incubated in almost the same method with the first extension. The product was purified in the same manner with the first extension. Finally, the second extension product was amplified by the ordinary PCR. Reaction mixture contained 10 μL of 5× PrimeSTAR buffer, 4 μL of 2.5 mM each dNTP mix solution, 2 μL of 10 μM NewLeft solution, 2 μL of 10 μM cnvKnewYtag solution, 1 μL of the second extension product solution, 0.5 μL of PrimeSTAR polymerase and 30.5 μL of UPDW. The incubation was performed in almost same manner with the second extension.

Other DNA samples including p53degΔK and p53degRand were prepared basically in the same protocol with the preparation of p53deg DNA. For p53degΔK, oligonucleotide GHis_cnvK was used instead of KHis_cnvK in the first extension. For the preparation of p53degRand, oligonucleotides p53_degRand and KHis_cnvK_short were used for the first extension and T7PURE_K was used in the second extension instead of T7PURE. Incubation program of the first extension was also modified as the annealing step was changed into incubating at 50°C for 15 s.

For the preparation of DNA samples of p53deg library and LX9 library for the actual selection, oligonucleotides named T7_PURE, p53deg_lib and p53deg_His_cnvK were connected into p53deg library and LX9_lib and LX9_His_cnvK were used instead of p53deg_lib and p53deg_His_cnvK respectively for LX9 library. In order to keep the library diversity, the primer overlap extension was performed in 100 μL scale for the libraries.

Overview of the model selection

Model selection using p53deg and its variants, p53degΔK and p53degRand, started from cDNA display construction. Each step of the display construction, including transcription, photo-crosslinking

reaction, translation and reverse transcription, was performed following the protocols described below. In order to see the ATP-dependency of the ubiquitylation of the displayed degron, two tubes of cDNA display samples of the original p53deg were prepared. The cDNA display samples were, then, subjected to *in vitro* ubiquitylation, his-tag purification. In order to confirm whether the ubiquitylation took place in an appropriate manner, the input, supernatant, wash and the eluent during his-tag purification were collected and analyzed by SDS-PAGE. The detailed procedure is described in "His-tag purification" section.

Because all ubiquitylated samples were used up for the PAGE analysis, the cDNA display samples of p53deg and p53degΔK were independently prepared for pulldown assay. The cDNA display samples were subjected to ubiquitylation and his-tag purification. In this time, no sample collection were done during the purification and all the eluents were subjected following antibody pulldown. qPCR assay was took place to evaluate whether the antibody can selectively pulldown the ubiquitylated p53deg (see "pulldown" and "qPCR assay" sections for detail).

Overview of the actual selection

In the two series of the actual selection, one of which started from p53deg library while the other of which started from LX9 library, most of procedures in the selection cycle were shared with the model selection. One of the differences is the amount of the applied mRNA in the initial round. As for p53deg library, 20 pmol of mRNA was used for the selection. It was accomplished by performing with four tubes in parallel while the model selection was performed with one tube for 10 pmol scale experiment. In the selection using LX9 library, 60 pmol of mRNA was converted into cDNA display and experienced ubiquitylation, his-tag purification, antibody pulldown and PCR amplification.

The selection was performed five rounds in total, including the initial round. After the final round, the initial library and the eluent from the final round were independently displayed, then subjected to ubiquitylation and antibody pulldown to evaluate the enrichment of the functional sequences. In this assay, two tubes of cDNA display sample were prepared for each DNA sample and one was subjected to ordinary ubiquitylation and the other was for ubiquitylation without ATP. Then recovery ratio was calculated both for the initial and final round's library by dividing the recovery in ATP-positive condition by that in ATP-negative condition. Since the selection can amplify undesired binders other than ubiquitylation substrates such as binders to streptavidin and beads scaffold, the recovery ratio, indicating the proportion of the actual functional sequences against background, is more suitable to evaluate the result of the selection rather than the direct recovery in ATP-positive condition. The experimental procedures were detailed in corresponding sections.

Transcription

DNA samples were transcribed into RNA using RiboMAX™ Large Scale RNA Production

Systems (Promega, product number: P1300). The reaction mixture of transcription consisted of 10 μL of 2× RiboMax buffer, 8 μL of template DNA solution and 2 μL of Enzyme Mix. After incubating the solution at 37°C for 30 min 1 μL of RQ1 DNase was added to the solution following additional incubation at 37°C for 15min. The product RNA was purified using 40 μL of RNAClean XP (Beckman Coulter, Inc., product number: A63987) magnetic beads as following the manufacturer's instruction. Finally, 40 μL of UPDW were added to the beads and mixed well to elute the RNA. Elution was done twice and both RNA solutions were mixed.

Photo-crosslinking reaction

The RNA samples underwent photo-crosslinking reaction to form covalent conjugates of RNA and puromycin linkers. The reaction mixture contained 5 μL of 2× cnvK buffer (100 mM Tris-HCl, 400 mM NaCl, pH 7.4), 1.2 μL of 10 μM RNA solution, 0.5 μL of 20 μM puromycin linker solution and 3.3 μL of UPDW. The mixture was headed at 90°C followed by 70°C for 60 s respectively. Then the temperature was decreased from 70°C to 25°C in velocity of 0.5°C/10 s to anneal the RNA and the linker. Finally, the mixture was incubated at 25°C for 1 min and stored at 10°C. Using Handy UV Lamp (As One, product number: LUV-6), 365 nm light was irradiated to the sample for 2 min.

Translation

RNA-linker conjugates were translated by PUREfrex1.0 system (GeneFrontier, product number: PF001-0.25). 12.5 μL of Solution I, 2.5 μL of Solution II and 2.5 μL of Solution III were added to 10 μL of photo-crosslinking product solution, mixed and incubated at 37°C for 1h. 12 μL of 3 M KCl solution and 3 μL of 1 M $MgCl_2$ solution were added to the reaction mixture and incubated at 37°C for another 1h. 10 μL of 0.5M EDTA (pH 8.0) solution was added and incubated at 37°C for 15 min. Subsequently, 50 μL of 2× SA binding buffer (10 mM Tris-HCl, 1 mM EDTA, 2 M NaCl, pH 7.5) was added.

Reverse transcription

Before carrying out reverse transcription, the translated RNA-linker conjugates, so it can be called mRNA display, were immobilized on SA beads by following protocol. 50 μL of SA beads (Dynabeads™ MyOne™ streptavidin C1, Invitrogen™, product number: 65001) was washed by 50 μL of 1× SA binding buffer three times. Translated sample was added to the beads and mixed by a mixer under room temperature for 30 min. Supernatant was discarded and the beads were washed with 50 μL of 1× SA binding buffer followed by 50 μL of 1× ReverTra Ace buffer.

Reverse transcription was done by ReverTra Ace system (TOYOBO, product number: TRT-101) on streptavidin beads. 10 μL of 5× ReverTra Ace buffer, 2 μL of 25 mM each dNTP mix and 1 μL of ReverTra Ace and 37 μL of UPDW were added to the beads, mixed well, then incubated at 42°C for

30 min. After the incubation, beads were washed with 50 μL of 1× Ubiquitylation buffer (50 mM Tris-HCl, 5 mM $MgCl_2$, pH 8.0) twice. 19.5 μL of 1× Ubiquitylation buffer and 0.5 μL of RNase T1 were added and incubated at 37°C for 15 min in order to elute cDNA display molecules from the beads.

Ubiquitylation

Ubiquitylation reaction mixture contained 3 μL of 10× Ubiquitylation buffer, 0.5 μL of 5 μM UBE1 (Recombinant Human His6-Ubiquitin E1 Enzyme (UBE1), R&D systems, product number: E-304-050), 1 μL of 25 μM UbcH5c (Recombinant Human UbcH5c/UBE2D3 Protein, R&D systems, product number: E2-627-100), 2.5 μL of 10 μM MDM2 (Recombinant Human MDM2/HDM2 Protein, R&D systems, product number: E3-204-050), 5 μL of 100 μM ubiquitin (Recombinant Human Ubiquitin Protein, R&D systems, product number: U-100H-10M), 0.5 μL of 100 mM DTT, 1.5 μL of 100 mM ATP and 20 μL of cDNA display solution and 12.5 μL of UPDW. The reaction mixture was incubated at 37°C for six hours in case for the model selection. In the ubiquitylation without ATP, the ATP solution in the mixture was simply replaced with UPDW.

In each round of the actual selection, the incubation time was changed to one hour.

His-tag purification

50 μL of Ni^{2+} supporting beads (His Mag Sepharose™ Ni, Cytiva, product number: 28967388) was washed with 50 μL of His binding buffer (20 mM phosphate buffer, 5 mM imidazole, 0.05% Tween 20, pH 7.4) once. 17 μL of the ubiquitylation reaction mixture was kept for SDS-PAGE and the remaining solution was added to the beads and incubated at room temperature overnight. After the incubation, 17 μL of supernatant was collected and the beads were washed by 50 μL of His tag binding buffer twice, then, product was eluted by 17 μL of His tag eluting buffer (1× PBS-T, 10 mM EDTA, pH 7.3) at room temperature for 30 min.

As for the pulldown assay of the model selection and each round of the actual selection, there were no intercepts of the sample during the purification and all the bound cDNA display were eluted by 40 μL of His tag eluting buffer.

Biotinylation of antibody

Anti-ubiquitin monoclonal antibody clone FK2 (Anti-Multi Ubiquitin mAb, MBL life science, product number: D058-3) was biotinylated using N-[[2-(biotinylamino)ethyl]dithiopropionyloxy]-sulfosuccinimide sodium salt (BSSO). First, 2.6 mg of BSSO was dissolved into 1 mL of PBS resulting. 100 μL of the antibody solution (1 mg/mL), 40 μL of PBS and 1.6 μL of BSSO solution were mixed well and incubated at 4°C for 1 hour. After the incubation, the biotinylated antibody was purified using Zeba Spin Desalting Columns, 7 KDa MWCO, 0.5 mL (Thermo Scientific, product number: 89882) to remove unreacted BSSO and hydrolyzed BSSO. The spin column was served on a collection tube

and centrifuged in 1500 g for 1 minute to remove storage solution. 300 μL of PBS-T was loaded on the resin and centrifuged in 1500 g for 1 minute. This washing process was repeated 3 times in total. The column was transferred onto a new tube and the crude sample of biotinylated antibody and additional 15 μL of PBS-T was applied onto the resin. The column was centrifuged in 1500 g for 2 minutes for elution.

In order to prepare the antibody conjugating beads, 20 μL of streptavidin (SA) beads was washed with 20 μL of PBS-T three times. The biotinylated antibody solution which containing 20 pmol of it and 10 μL of PBS-T was applied to the beads and incubated at 4°C for 1 hour. The supernatant was discarded, and the beads were washed with 20 μL of PBS-T.

Antibody pulldown

50 μL of streptavidin beads were washed with 50 μL of PBS-T three times and the eluent from his tag purification was applied the beads with adding 60 μL of PBS-T. The sample was incubated at room temperature for 1 hour for negative selection. After the incubation, 10 μL of the supernatant was collected for qPCR. 20 μL of antibody beads and 20 μL of streptavidin beads served for pulldown and 54 μL from remaining 110 μL of supernatant of previous incubation was added to both beads. The beads were incubated at 4°C overnight, and the supernatants were collected. Both beads were washed with 100 μL of PBS-T containing NaCl in concentration of 0.5 M. The washing was repeated three times. 100 μL of 50 mM Tris-HCl buffer (pH 7.0) containing TCEP in concentration of 10 mM were loaded and mixed at room temperature for 1 hour. The eluent was collected and used for qPCR.

As for the actual selection, the negative selection was performed in the same protocol with one described above, however the collected supernatant was incubated with 20 pmol of biotinylated antibody rather than the antibody beads. Then the complex of ubiquitylated cDNA display and the biotinylated antibody was pulled down using 50 μL of the naked streptavidin beads. The incubation condition was also changed to 5 minutes at room temperature. The following washing step was skipped in the initial round of the selection. In the 2nd and 3rd round, beads were washed with the NaCl containing Tris-HCl buffer once and twice respectively before the elution. For 4th and 5th round, the washing was performed three times. The reductive elution was performed following the same protocol with model selection.

PCR amplification

In the actual selection, the eluent of the pulldown was purified by the FavorPrep purification kit, then subjected to PCR amplification in order to get a DNA library for the next round using PrimeSTAR HS DNA Polymerase system. 100 μL of reaction mixture contained 20 μL of 5×PrimeSTAR buffer, 8 μL of 2.5 mM each dNTP mix solution, 5 μL of 10 μM T7PURE_short solution, 5 μL of 10 μM cnvK_newYtag solution, 1 μL of PrimeSTAR polymerase, 40μL of the purified eluent and 21 μL

UPDW. The program of the thermal cycler was the same with that of the PCR amplification in the library preparation. After the reaction, the amplified DNA was purified using the FavorPrep purification kit in the same protocol described above and subjected to the next round selection. The selection was continued five times in total.

qPCR assay

The collected samples during the pulldown procedure were purified by FavorPrep™ spin column in the same protocol with the purification of DNA The purified cDNA display samples were subjected to qPCR using THUNDERBIRD Probe qPCR Mix (TOYOBO, product number: QPS-101) and StepOne™ Real-Time PCR System (Applied Biosystems™, product number: 4376374). The reaction mixture contained 10 μL of qPCR Mix, 0.6 μL of 10 μM forward primer solution, 0.6 μL of 10 μL reverse primer solution, 1 μL of EvaGreen (Biotium, Inc. product number: 31000), 0.4 μL of ROX dye, 5.4 μL of UPDW and 2 μL of cDNA display sample. The oligo nucleotide p53_degqPCR_L was used as forward primer for p53deg and p53degΔK in common. As for the reverse primer, p53_degqPCR_R was used for p53deg and another oligo nucleotide p53_degqPCR_RΔK was used for p53degΔK. For p53deg library and LX9 library, primer pairs of p53lib_qPCR_F, p53lib_qPCR_R and LX9_qPCR_F, LX9_qPCR_R were used respectively.

Based on the measured cDNA concentrations in the supernatant of the first negative selection and the eluents of the positive selection, cDNA display recovery for p53deg and p53degΔK was calculated using following formula. Because 54 μL of the supernatant were subjected to the positive selection while the concentration was measured using 10 μL of the sample, the numerical coefficient 5.4 is multiplied to the concentration of the supernatant of negative selection.

$$\text{Recovery}[\%] = \frac{\text{cDNA in the eluent of positive selection}}{5.4 \times \text{cDNA in the supernatant of negative selection}} \times 100$$

For the two libraries of the actual selection, the recovery ratio of each kind of samples were calculated by dividing the recovery in ATP positive condition by that in ATP negative condition as follows.

$$\text{recovery ratio} = \frac{\text{recovery in ATP positive condition}}{\text{recovery in ATP negative condition}}$$

Next-generation sequencing

Next-generation sequencing was carried out using MiSeq System (Illumina, Inc., product number: SY-410-1003) and MiSeq Reagent Kit v3 (Illumina, Inc., product number: MS-102-3001).

Before carrying out the run, the DNA sample was amplified by two rounds of PCR to add sequences required for the sequencing including the index sequence and priming region for the bridge PCR. For the LX9 library samples, 50 μL of reaction mixture of the first round contained 10 μL of 5×PrimeSTAR buffer, 4 μL of 2.5 mM each dNTP mix solution, 2.5 μL of 10 μM Rd1SP_LX9 solution, 2.5 μL of 10 μM Rd2SP_common solution, 0.5 μL of PrimeSTAR polymerase, 0.5 μL of DNA template and 30 μL UPDW. The Rd1SP_LX9 solution was replaced with Rd1SP_p53deg in case of the p53deg library samples. The program of the thermal cycler was basically the same with the PCR amplification, but the incubation time of step 4 was changed to 9 seconds and the number of cycles was eight. The DNA sample was purified using FavorPrep spin column in the same protocol described above. In the second round, 2.5 μL of 10 μM appropriate index primers were added instead of Rd1SP_LX9 or Rd1SP_p53deg, and Rd2SP_common. The program of the thermal cycler was the same with that in the first round. The product was purified again, then the DNA sample was diluted into 4 nM solution by 10 mM Tris-HCl (pH 8.5) containing 0.1% Tween 20.

In the next step, the DNA sample was denatured by following protocol. First, 5 μL of the 4 nM DNA solution and 5 μL of 0.2 M NaOH solution were mixed and incubated five minutes at room temperature. 990 μL of iced Hyb buffer was added to get a 20 pM denatured DNA sample. 4 nM PhiX control DNA was denatured in the same manner. Then, 400 μL of Hyb buffer, 200 μL of the 20 pM denatured DNA sample and 200 μL of the 20 pM denatured PhiX were mixed.

Finally, 600 μL of the denatured sample was added to the reagent cartridge and set to the machine. Running procedure was done by following the instruction from the MiSeq's built-in control software.

BLAST analysis

The Protein BLAST online interface (https://blast.ncbi.nlm.nih.gov/Blast.cgi) was utilized to search for the top four sequences containing lysine and the top four sequences lacking any lysine residues. The aligned entries were then manually scrutinized to identify animal proteins possessing similar sequences to the discovered motifs.

List of primer sequences

T7PURE

GATCCCGCGAAATTAATACGACTCACTATAGGGAGACCACAACGGTTTCCCTCTAGAAAT
AATTTTGTTTAACTTTAAGAAGGAGATATACCAATG

T7PURE_K

GATCCCGCGAAATTAATACGACTCACTATAGGGAGACCACAACGGTTTCCCTCTAGAAAT
AATTTTGTTTAACTTTAAGAAGGAGATATACCAATGAAA

T7PURE_short

GATCCCGCGAAATTAATACGACTCACTATAGGGAGACCACAACGGTTTCCCTCTAG

p53_deg

GTTTAACTTTAAGAAGGAGATATACCAATGAAACCGCTTTCATCTTCCGTCCCCTCTCAAA
AGACTTATCAGGGGTCATACGGCTTTCGTCTTGGGAA

p53_degΔK

GTTTAACTTTAAGAAGGAGATATACCAATGGCACCGCTTTCATCTTCCGTCCCCTCTCAAG
CAACTTATCAGGGGTCATACGGCTTTCGTCTTGGG

p53_degRand

AAGGAGATATACCAATGAAANNKNNKNNKNNKNNKNNKNNKNNKNNKNNKNNKNNKN
NKNNKNNKNNKNNKNNKNNKNNKNNKAAGAAGAAGAAGGGTGG

p53deg_lib

AACTTTAAGAAGGAGATATACCAATGAAGAAGAAGCCCCTGTCATCTTCTGTCCCTTCCC
AGNNKNNKNNKNNKNNKNNKNNKNNKTTCCGTCTGGGCAA

LX9_lib

GTTTAACTTTAAGAAGGAGATATACCAATGGCAGGTGGTTCTNNKNNKNNKNNKNNKNN
KNNKNNKNNKGGCGGCAGCGGTGGAGGAAGTCATCATCAC

GHis_cnvK

TTTCCACGCCGCCCCCCGTCCTTGAGCCTCCATGGTGATGGTGGTGGTGGGAGCCCCCAC
CTGAGCCTCCTGCCCCAAGACGAAAGCCGTA

KHis_cnvK
TTTCCACGCCGCCCCCCGTCCTTGAGCCTCCATGGTGATGGTGGTGGTGGGAGCCCCCAC
CTTTTTTTTTCTTCCCAAGACGAAAGCCGTA

KHis_cnvK_short
TTTCCACGCCGCCCCCCGTCCTTGAGCCTCCATGGTGATGGTGGTGGTGGGAGCCCCCAC
CCTTCTTCTTCTT

p53degHis_cnvK
TTTCCACGCCGCCCCCCGTCCTTGAGCCTCCATGGTGATGGTGGTGGTGACTTCCTCCAC
CCTTCTTCTTGCCCAGACGGAA

LX9His_cnvK
TTTCCACGCCGCCCCCCGTCCTGCTGCCGCCACCATGGTGGTGGTGATGATGACTTCCTC
CACC

NewLeft
GATCCCGCGAAATTAATACGACTCACTATAGGG

cnvK_NewYtag
TTTCCACGCCGCCCCCCGTCCT

p53deg_qPCR_L
TCCCGCGAAATTAATACGAC

p53deg_qPCR_R
TTTCTTCCCAAGACGAAAGC

p53degΔK_qPCR_R
AAGCCGTATGACCCCTGATA

p53lib_qPCR_L
GGGAGACCACAACGGTTTC

p53lib_qPCR_R
GGTGATGGTGGTGGTGACTT

LX9_qPCR_L
ACCACAACGGTTTCCCTCTA

LX9_qPCR_R
CGTGATGATGGTGATGATGG

Rd1SP_p53deg
CACTCTTTCCCTACACGACGCTCTTCCGATCTCCCCTGTCATCTTCTGTCCC

Rd1SP_LX9
CACTCTTTCCCTACACGACGCTCTTCCGATCTATATACCAATGGCAGGTGGTTC

Rd2SP_common
GACTGGAGTTCAGACGTGTGCTCTTCCGATCTTTTCCACGCCGCCCCCCG
